# Leveraging human capital to reduce maternal mortality in India: enhanced public health system or public-private partnership?

**DOI:** 10.1186/1478-4491-7-18

**Published:** 2009-02-27

**Authors:** Karl Krupp, Purnima Madhivanan

**Affiliations:** 1 Public Health Research Institute, Yadavgiri, Mysore, India; 2 San Francisco Department of Public Health, San Francisco, CA, USA

## Abstract

Developing countries are currently struggling to achieve the Millennium Development Goal Five of reducing maternal mortality by three quarters between 1990 and 2015. Many health systems are facing acute shortages of health workers needed to provide improved prenatal care, skilled birth attendance and emergency obstetric services – interventions crucial to reducing maternal death. The World Health Organization estimates a current deficit of almost 2.4 million doctors, nurses and midwives. Complicating matters further, health workforces are typically concentrated in large cities, while maternal mortality is generally higher in rural areas. Additionally, health care systems are faced with shortages of specialists such as anaesthesiologists, surgeons and obstetricians; a maldistribution of health care infrastructure; and imbalances between the public and private health care sectors. Increasingly, policy-makers have been turning to human resource strategies to cope with staff shortages. These include enhancement of existing work roles; substitution of one type of worker for another; delegation of functions up or down the traditional role ladder; innovation in designing new jobs;transfer or relocation of particular roles or services from one health care sector to another. Innovations have been funded through state investment, public-private partnerships and collaborations with nongovernmental organizations and quasi-governmental organizations such as the World Bank. This paper focuses on how two large health systems in India – Gujarat and Tamil Nadu – have successfully applied human resources strategies in uniquely different contexts to the challenges of achieving Millennium Development Goal Five.

## Review

Recently the association between human resources (HR) and population health has received considerable attention. There is growing evidence that HR inputs are an important determinant of broader population-based outcomes such as maternal mortality [1]. The issue is of crucial importance to developing countries facing the triple threat of rising demand, escalating costs and human resource shortages in public health care systems. This paper will use India as a lens to examine the broader issues surrounding human resources and public health. It will explore some of the HR strategies employed in a variety of settings with mixed results. Finally, it will look at several very contrasting approaches employed by two Indian states, Tamil Nadu and Gujarat, in dealing with human resource shortages as they struggle to reduce maternal mortality.

## Background

Each year, roughly 27 million women give birth in India [2]. Of these, about 136 000 die as a direct result of their pregnancy and delivery [3]. India accounts for more than 20% of the global burden of maternal mortality and the largest number of maternal deaths for any country [4]. Most of these deaths are caused by haemorrhage (29%), anaemia (19%), sepsis (16%), obstructed labour (10%), unsafe abortion (9%) and hypertensive disorders of pregnancy (8%) [5].

The relationship between lack of pregnancy-related care and maternal death is well recognized [6]. It is widely believed that most maternal mortality is preventable with skilled obstetric care [7,8]. The World Health Organization (WHO) has prioritized skilled birth attendance (SBA) as a critical strategy for reducing maternal mortality in developing countries [9]. WHO defines SBA as "accredited health professional(s) – such as a midwife, doctor or nurse – who has been educated and trained to proficiency in the skills needed to manage normal (uncomplicated) pregnancies, childbirth and the immediate postnatal period, and in the identification, management and referral of complications in women and newborns" [10].

Currently there is a worldwide shortage of almost 4.3 million practitioners meeting the WHO definition [11]. In countries like India, 46.6% of births are attended by an SBA [12] but skilled attendance in rural areas is as low as 33.5% [13]. Not surprisingly, studies in India have confirmed the importance of SBAs, showing an inverse relationship between distribution of trained birth attendants and maternal mortality ratios [14].

In the aggregate, India has human resources for health comparable to other low-income countries. With seven physicians and eight nurses per 10,000 population, the country compares favorably with Pakistan, for instance, which has 7.4 doctors and 4.7 nurses per 10,000 population [15,16]. What aggregate numbers fail to capture, however, is that India is one of the most privatized medical systems in the world. The public health care system, which provides the only health care access for the poor, has only two physicians and eight nurses per 10,000 population [15]. This human resource shortfall extends across all categories in the system, including shortages of female health assistants (30%), specialized doctors (68%), nurses and midwives (41%), and radiographers (57%) [17].

Complicating the human resource picture further, the government of India has vacillated widely on initiatives to train SBA. In the 1960s, midwives were trained in large numbers to provide maternal and child health services. After 1966, with pressure from international agencies, their role shifted from midwifery to family planning and immunization [18]. At the same time, institutional midwives were replaced with general nurses and midwife training was eliminated. As a consequence, while many nurses are currently classified as midwives, few have the skill sets required to qualify as SBAs [18].

For India to meet the Millennium Development Goal of reducing maternal deaths by 75% from 1990 levels, the maternal mortality ratio (MMR) will have to be reduced to 109 per 100,000 live births from the current level of 301 per 100,000 live births [19]. Based on current trends, an MMR of 160 is predicted for 2015 [20]. Given that shortfall, both the central and state governments are aggressively looking for ways to achieve further reductions in spite of current human resource shortages.

### Human resources – a crucial input to health systems

There is an emerging consensus that a lack of financial resources explains only part of the slow progress towards improved health indicators made by most developing countries [21]. In India, a little more than 73% of all health spending is out-of-pocket, 6% from third-party insurers and employers, and the remainder from government [22]. States typically account for about two thirds of these public expenditures, and the central government the remaining one third [23].

The largely privatized nature of the spending has contributed to huge inequities among the states. In 2005, for instance, overall health spending in Himachal Pradesh, at USD 98 per capita, was almost five times Tamil Nadu's annual health expenditure, at USD 20 per person.

Interestingly, spending levels appear to have only the most general correlation with health indicators. In 2005, Tamil Nadu's infant mortality rate (IMR) was 9% lower than that of Himachal Pradesh; under-four mortality was 31% lower, and life expectancy was 3.4 years longer (Table 1).

**Table 1:** 2005 expenditures on health for selected states of India

**State**	Overall spending per capita (USD)*^1^	**Public spending per capita (USD)**^**1**^	Infant mortality rate (2005)**^2^	Average life expectancy (2005)**^2^	Child mortality among 0–4 years (2005)**^3^
Himachal Pradesh	98.18	12.17	36	65	13.5%

Kerala	73.80	7.97	15	73.3	3.4%

Jammu & Kashmir	52.05	10.77	45	63	12.0%

Punjab	45.33	8.16	42	70.9	11.3%

Haryana	44.65	4.73	42	67	17.8%

Maharashtra	39.40	8.71	38	68.3	8.6%

Bihar	37.43	3.11	62	65.2	20.1%

Assam	33.68	5.99	66	59.9	19.7%

Madhya Pradesh	30.00	4.08	70	58.6	24.6%

West Bengal	29.70	5.14	48	67.7	10.0%

Gujarat	29.68	4.69	50	63.6	16.0%

Uttar Pradesh	28.80	3.74	73	63.8	24.7%

Andhra Pradesh	27.95	5.42	53	63.9	14.8%

Karnataka	24.93	5.78	43	64.4	13.1%

Tamil Nadu	23.33	6.20	31	68.4	9.0%

*1 USD = 40 INR, ** From

^1^Economic Research Foundation. Government Health Expenditure in India: A Benchmark Study. August 2006 .

^2^State Level Tables. Human Development Report 2007. Andhra Pradesh .

^3 ^Government of India. India and State wise Child Mortality Rate (0–4 years) .

How can we explain these differences in health indicators, given the enormous disparity in resources? There is growing evidence that health system components (e.g. financing, human resources and governance) determine in large part the success or failure of health systems [24]. Among these, management of human resources has been cited as the most crucial factor for success of developing country health systems [25].

WHO, in its *World health report 2000*, identified three principal health system inputs: human resources (HR), physical capital and consumables [26]. While each of these is important to the delivery of health services, HR is critical to the success of any health system. Put simply, the ultimate impact of any health programme hinges on whether health care workers actually deliver those services. Not surprisingly, human capital is one of the largest assets available within a health system and is frequently the single greatest expense in any national health care budget. In many countries it represents as much as two thirds of the total recurring costs [26].

In spite of its central position in health care systems, HR typically receives less attention than investment in buildings and technology. Since 1951 the government of India has focused heavily on capital infrastructure without any comparable investment in human capital. While the country's rural health system is impressive, with almost 146,000 subcentres, 23,000 primary health centres (PHCs) and just over 3,000 community health centres (CHCs), shortages of human resources are apparent at every level [27]. More than 7% of subcentres operate without an auxiliary nurse midwife (ANM) and 50% without a male health worker [28]. More than 800 PHC have no physician [17], and CHCs face deep shortages of obstetricians and gynaecologists (56%), paediatricians (67%) and surgeons (56%) [27].

Unfortunately, in today's increasingly globalized world, many HR challenges have moved beyond the control of individual health care systems. India is not untypical in facing a crisis of emigration of doctors and nurses to Australia, Canada, the United Kingdom and the United States of America. Among developing countries, it is one of the largest exporters of health care professionals, with India-trained physicians accounting for approximately 4.9% of practising physicians in the United States, and 10.9% in the United Kingdom [29]. One study estimated that almost 11% of graduates for all medical schools in India emigrated to other countries to practise [29]. The situation is similar for nurses. A recent survey carried out at two large nursing schools in India showed that approximately 50% of graduating students migrate out of the country [30]. This has huge implications for staffing and training within the public health system. Studies have shown that India has lost up to USD 5 billion in training costs since 1951 because of emigration [31].

### Human resources and maternal mortality

Researchers exploring the linkages between human resources and maternal mortality have reached contradictory findings. Robinson and Wharrad [32,33] showed that density of doctors was significantly related to maternal outcomes. In contrast, Cochrane et al. reported that physicians per capita had no effect on maternal mortality [34]. Similarly, neither Kim and colleagues nor Hertz et al. found a significant association between doctor density and maternal death [35,36]. Most recently, Anand and Bärnighausen, using new data from WHO, found a strong negative correlation between the concentration of physicians and maternal mortality [1]. Interestingly, all six studies showed no association between nurse density and improvement in maternal outcomes.

Given the conflicting data, what is the takeaway lesson about physician density and its relationship to maternal mortality? While all the studies have strengths and weaknesses; Anand and Bärnighausen's analysed newer WHO data from 198 countries and is the largest and most comprehensive to date. Their findings suggest that doctors appear best able to address the largest proportion of conditions putting mothers at risk. In addition, such a conclusion would also be consistent with findings showing that developing countries with a shortage of doctors but a large cadre of nurses have had more success with lowering under-five mortality, a health care challenge requiring less specialized interventions, than they have with lowering maternal mortality [1].

### Strategies to leverage existing human resources

Since it seems likely that emigration of physicians and nurses will be a continuing problem, given the low salaries and poor working conditions in developing countries, how can policy-makers address shortages and skill-mix discontinuities? Sibbald and colleagues, in a recent literature review, suggest seven strategies that have been used to realign human resources in health systems [37]:

• Enhancement: upgrading a particular job by increasing the skill level of workers or enhancing the role with additional responsibilities;

• Substitution: exchanging one type of worker for another. This might mean for instance, training nurses to take on the role of doctors in primary health care delivery;

• Delegation: moving particular tasks up or down a traditional role ladder;

• Innovation: creating new jobs by introducing a new type of worker with a different role;

• Transfer: moving particular jobs from one health care sector to another;

• Relocation: shifting particular services from one healthcare sector to another;

• Liaison: using specialists in one health system sector for support workers in another.

Developing countries have tried all these strategies, with mixed results. During the 1970s and 1980s, traditional birth attendants (TBA) were trained in midwifery (enhancement) but this appeared to have little impact on maternal outcomes [38]. While there is evidence from developing countries that appropriately trained nurses can replace doctors in many care settings (substitution) [39], previously mentioned econometric studies throw serious doubt on whether this strategy is effective in other settings – particularly in developing countries, where nurse and midwife training is often inadequate [1].

The use of TBAs in managing postpartum haemorrhage using the drug Misoprostol has been documented in several resource-poor countries [40,41]. Since this traditionally would be carried out by a doctor or trained nurse, this task has been shifted down the role ladder (delegation).

There have also been efforts to create new categories of workers (innovation). One particularly successful example is the use of lay health workers to promote immunization and improve outcomes for acute respiratory infections and malaria [42].

There have been a variety of efforts to transfer primary health care functions and sometimes even government staff (transfer/relocation), from the public sector to nongovernmental organizations and private providers when there was a critical need for additional capacity [43]. Finally, government health care workers have been used extensively in Africa and Asia to train and support private practitioners [44], an example Sibbald et al. would label a "liaison" strategy.

Considering the scope of the problem, surprisingly little attention has been given to HR management in India. Most efforts have been focused on pilot projects using community health workers in HIV education and testing [45], child nutrition and survival [46], pneumonia management [47] and malaria screening and treatment [48]. While some efforts have shown promise, sustainability has been poor because of limited funding from external sponsors. More recently, the government has been experimenting with community health workers called "accredited social health activists" (ASHA) to carry out a variety of health initiatives as part of the National Rural Health Mission [27], but the impact of this strategy is not yet clear. In contrast, on the state level there are a number of innovative and successful programmes realigning human resources, some even decades old. This paper will focus on two very different approaches successfully employed by the states of Gujarat and Tamil Nadu to realign human capital and reduce maternal mortality.

### Relocating obstetric gynaecology services from the public to private sector in Gujarat

Gujarat, one of India's leading industrial states, is located on the western tip of the country. Despite its ranking among the top five states in the country in per capita income, social and health indicators have lagged far behind those of many of its less well-off neighbours. In 2005, the state had an MMR of 172 per 100 000 live births. While that number was lower than the all-India MMR of 301, it still came in well above Kerala and Tamil Nadu, at 110 and 134, respectively [49]. In that year, the state also had an infant mortality rate (IMR) of 54 per 1000 births, almost on par with the all-India average of 58. In contrast, Kerala had an IMR of 14, Maharashtra 36, Tamil Nadu 37, West Bengal 38, and Uttaranchal 42[50].

With those grim statistics in mind, Gujarat set out in 2005 to lower maternal and infant mortality. The primary obstacle to the state's efforts was a shortage of human resources. Shockingly, there were only seven public sector obstetrician/gynaecologists (OB/GYN) providing services to a rural population of almost 32 million. In contrast, Gujarat had more than 700 private OB/GYN practising in rural areas. The disparity is not surprising, since private sector specialists receive salaries typically five times higher than those earned in comparable positions in government service [51]. Following a series of consultations with both public and private stakeholders, the government developed a Public Private Partnership (PPP) called "Chiranjeevi Yojana" which realigned health system human resources by relocating obstetric gynaecology services from the public sector to the private sector in Gujarat [52].

The scheme was first pilot-tested in five predominantly rural districts, and then scaled up across the state. Under the scheme, the Gujarat Health & Family Welfare Department recruited providers who had postgraduate qualifications in obstetrics and gynaecology; owned their own hospital with a labour room, operating theatre and blood bank; and had access to anaesthesiology services. In return, the state reimbursed physicians approximately USD 40 per delivery. Rather than pay providers directly, the Chiranjeevi Yojana scheme distributed vouchers to all pregnant women living below the poverty line (approximately USD 9 to USD 14 per person per month). Eligible women could choose a local OB/GYN and exchange the voucher for delivery services, free medicines and transport reimbursement [52,53].

Through November of 2007, "Chiranjeevi Yojana" enrolled 843 providers and provided for almost 143,000 deliveries. While 642 maternal deaths might have been anticipated in the programme through then, only 31 were reported. Strikingly, only 454 infants died, against an expectation of 6561 in the absence of the programme. Even more impressive, Gujarat was able to deliver these results through the direct relocation of obstetric gynaecology services from public to the private sector [52].

### Using human resource strategies to address health worker shortages in Tamil Nadu

Tamil Nadu is the eleventh largest state in India by area, and the sixth most populous. When compared with All-India statistics, the health status of residents of Tamil Nadu is considerably above average and has seen significant improvement over the years [54]. Infant mortality rates have declined from 37 per 1000 in 2005 to 31 per 1000 in 2005/2006 – considerably lower than the All-India rate of 57 per 1000. The state has also made dramatic improvements in maternal mortality, reducing MMR from 195 per 100,000 live births in 1996 to 71 per 100 000 live births in 2007 [54,55].

In contrast to Gujarat's almost exclusive reliance on PPP, Tamil Nadu has continued to champion a public primary health care model while still struggling with many of the same challenges plaguing other areas of India. For some years, the state has faced chronic shortages of surgeons, anaesthesiologists, obstetrician gynaecologists and laboratory technicians in the public health system [56]. In spite of that, the government has continued to invest in health infrastructure, including new primary health centres (PHCs) and extended hours at existing centres [57]. In order to deal with staff shortages, the state has successfully used a variety of HR strategies, including enhancement of the non-specialist physician and nursing roles, innovations such as the creation of Comprehensive Emergency Obstetric Newborn Care Centres (CEmONC) in 51 government hospitals [58], and the relocation of some health system functions to the private sector.

As part of its effort to change the skill mix of its workforce, the Government of Tamil Nadu has been aggressively enhancing the roles of non-specialist physicians and nurses. Doctors with MBBS degrees, the lowest qualification for an allopathic physician, are being trained in surgery, obstetrics, anaesthesia and radiology [59,60] in order to cope with shortages of specialists. There has also been a concerted effort to upgrade the skills of staff nurses with training in first aid, use of Misoprostil to prevent postpartum haemorrhage, maternal administration of magnesium sulfate, and better birthing practices [61]. Additionally, laboratory technicians are being credentialed as X-ray technicians to increase diagnostic capability at PHCs [61].

Tamil Nadu has also focused on reorganizing its public health care systems to ensure accessibility of emergency obstetric care. CEmONC have been established at two hospitals in each district and staffed with specialists in ob/gyn, paediatrics, anaesthesia and general surgery in order to provide referral emergency obstetric and newborn care services 24 hours a day, seven days a week. Each CEmONC has an operating theatre, blood bank, diagnostic laboratory and ambulance service. At current levels, mothers from any area in Tamil Nadu can gain access to emergency obstetric care within one hour [62].

Tamil Nadu has also been encouraging public-private partnerships to facilitate the provision of ancillary services. While the state continues to provide most medical care, it is experimenting with private sector collaborations for ambulance services, facility maintenance, medical equipment, sanitation and construction, to name just a few [63,64]. In addition, Tamil Nadu is establishing PPPs to provide health care access in tribal areas. Presently it has collaborations with the private companies and NGOs for mobile outreach clinical services, blood banks and provision of training and support for community health workers in remote areas [64].

While overall maternal mortality continues to decline in Tamil Nadu, there is a dearth of data on the impact of individual health system strengthening measures on maternal mortality. The state is currently developing an online monitoring and evaluation system to provide real-time data on health system inputs, outputs and impact [65]. Once in place, the system should provide additional information on how various initiatives will affect population-based health indicators such as MMR. As part of these efforts, there is a compelling need for additional research into the contribution of human resource strategies in reducing maternal death in Tamil Nadu.

## Conclusion

With the current acute shortage of health care workers in developing countries, it has never been more urgent to assess how different human resource levers might be used to improve population-based health outcomes. It is telling that Gujarat and Tamil Nadu – the states which are among the most aggressive in experimenting with HR strategies – are also among the top performers in reducing maternal and neonatal mortality in India. The experience of both states however, shows that there is no single recipe for success.

Gujarat was able to effectively relocate the obstetrician gynaecologist role from the public sector to the private sector because there were sufficient numbers of specialists practising in rural areas. Unfortunately, in many states where maternal mortality is problematic, most OB/GYNs practise in urban centres. Similarly, Tamil Nadu's public health infrastructure, while somewhat neglected, has historically been among the best in India. In this context, investing in enhanced maternal care made sense, given the already extensive infrastructure available. Perhaps the main lesson that can be taken from both examples is that solutions need to be homegrown, since context often provides both obstacles and opportunities for productive change.

The examples also seem to confirm the critical nature of certain human resource inputs, in particular skilled surgical and obstetric care. Interventions also require the presence of physical infrastructure – operating theatres, blood banks, diagnostic laboratories and emergency transportation – in order to realize the benefits of investment in human capital. Gujarat was able to leverage private resources and avoid heavy investment in bricks and mortar, while Tamil Nadu had a strong foundation for continued investment. Whether either lesson is of value to other health systems will depend almost entirely on the circumstances of the beholder.

Achieving Millennium Development Goal Five – reducing maternal deaths and providing universal access to reproductive health – will require substantial health system reform in many developing countries. Most, like India, face acute human resource shortages – particularly in rural areas where the needs are often greatest. In order to be successful, policy-makers will have to leverage a wide spectrum of resources, both public and private, to address the health needs of their populations. Realigning human resources through thoughtful use of public private transfer, task shifting, and position enhancement may offer the best opportunity for achieving improved health outcomes for women and children in resource-constrained settings.

## Competing interests

The authors declare that they have no competing interests.

## Authors' contributions

KK and PM conceived the paper. KK drafted the outline, the problem statement and conclusions. PM reviewed and edited the whole manuscript. Both authors contributed to the reference search and read and approved the final manuscript.
